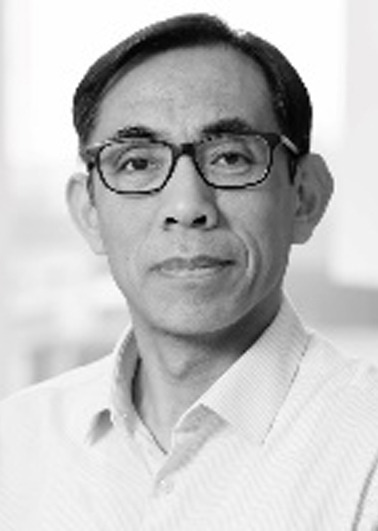# Modelling Development in Radical (Co)Polymerization of Multivinyl Monomers

**DOI:** 10.1002/anie.202212235

**Published:** 2022-12-14

**Authors:** Jing Lyu, Yinghao Li, Zishan Li, Piotr Polanowski, Jeremiasz K. Jeszka, Krzysztof Matyjaszewski, Wenxin Wang

**Affiliations:** ^1^ Charles Institute of Dermatology School of Medicine University College Dublin Dublin Ireland; ^2^ Department of Molecular Physics Technical University of Lodz 90-924 Lodz Poland; ^3^ Department of Mechanical Engineering Informatics and Chemistry of Polymer Materials Technical University of Lodz 90-924 Lodz Poland; ^4^ Center for Macromolecular Engineering Department of Chemistry Carnegie Mellon University Pittsburgh PA 15213 USA; ^5^ School of Mechanical and Materials Engineering University College Dublin Dublin Ireland; ^6^ School of Public Health Anhui University of Science and Technology Huainan China

**Keywords:** Gelation Process, Modelling, Monte Carlo, Multivinyl Monomer, Radical (Co)Polymerization

## Abstract

Radical polymerization (RP) of multivinyl monomers (MVMs) provides a facile solution for manipulating polymer topology and has received increasing attention due to their industrial and academic significance. Continuous efforts have been made to understand their mechanism, which is the key to regulating materials structure. Modelling techniques have become a powerful tool that can provide detailed information on polymerization kinetics which is inaccessible by experiments. Many publications have reported the combination of experiments and modelling for free radical polymerization (FRP) and reversible‐deactivation radical polymerizations (RDRP) of MVMs. Herein, a minireview is presented for the most important modelling techniques and their applications in FRP/RDRP of MVMs. This review hopes to illustrate that the combination of modelling and wet experiments can be a great asset to polymer researchers and inspire new thinking for the future MVMs experiment optimization and product design.

## Graphical abstract

The major developments of modelling approaches in radical polymerization of multivinyl monomers (MVM) are summarized and their applications on FRP and RDRP are highlighted. This Minireview hopes to show that the combination of modelling and wet experiments can be a great asset to polymer scientists. It aims to serve as a concise introduction to push the boundary of using modelling techniques to understand the mechanism and product structure in MVM polymerization.

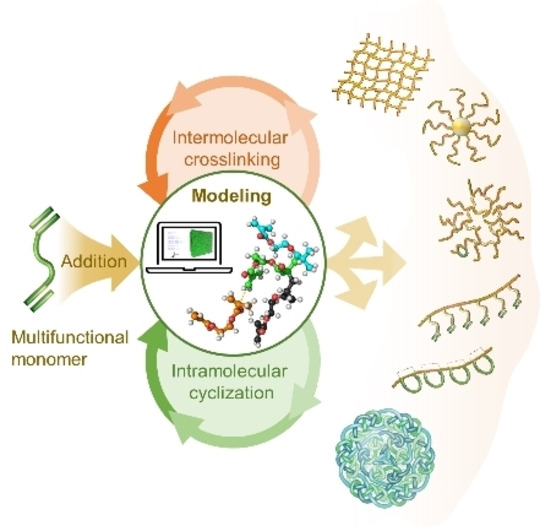

## 1 Introduction

Architecturally complex polymers have attracted a lot of attention in both the industrial and polymer scientific fields due to their well‐defined structure and specific functionality. To manipulate polymer topology, it is necessary to incorporate multivinyl monomers (MVMs) in polymerization processes to involve multiple reactive groups.^[1]^ The radical‐based process of MVMs (and monovinyl monomers) is one of the most widely used chemical methods for synthesis of free radical polymerization (FRP) thermosets.^[2]^ Currently, the conventional FRP contributes annually to half of the worldwide production of synthetic polymers^[3]^ (closer to 40 % now) though it is not able to yield the controlled molecular weight (MW), molecular weight distribution (MWD) or the block copolymer structure.^[4]^ The reversible‐deactivation radical polymerization (RDRP) is an advantageous method to prepare well‐controlled topologies. In RDRP, the establishment of the fast dynamic equilibrium between active radicals and dormant species extends the lifetime of growing radicals ensuring the simultaneous propagation of all propagating chains.^[5]^ Three most promising types of RDRP are nitroxide mediated polymerization (NMP),^[6]^ atom transfer radical polymerization (ATRP)^[7]^ and reversible addition fragmentation chain transfer polymerization (RAFT).^[8]^

**Figure 1:**
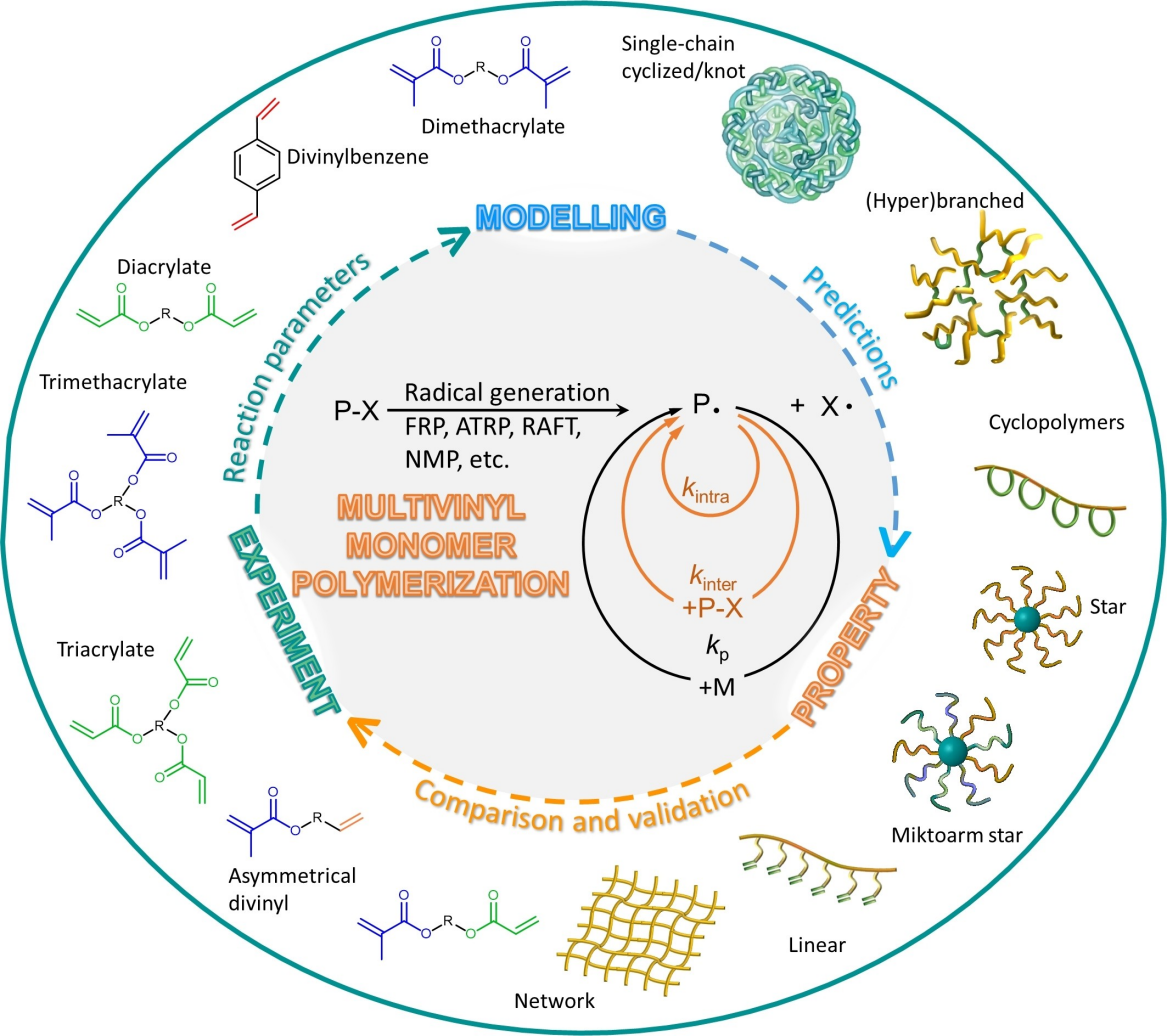
Illustration of the relationship among experiment, modelling, and polymer properties.

To date, however, inadequate mechanism understanding of the MVMs polymerization systems has been one of the key obstacles to expanding the application spectrum of these polymerization strategies. Owing to the inherent limitations of the experimental characterizations, it is rather difficult to obtain the instantaneous reaction parameters and precise structure information at each instant and to characterize soluble products from rapid gelation processes. From this point of view, modelling technique has become a powerful tool for the study of MVMs polymerization. Modelling techniques contribute complementary information which is not easily accessible through experiments, especially those involving branching and gelation (such as the extent of intramolecular cyclization and intermolecular crosslinking, radical concentration, dead chain fraction etc.).^[9]^ Such information is useful for optimizing experimental conditions, tailoring polymer architecture and tuning their properties.[^2^, ^10^] In the meantime, modelling cannot be separated from experiments. For the sake of simplicity, models are usually established with simplifying assumptions, which should be consistent with the reaction conditions, and the modelling results must be validated by the experimental data (Figure 1).

In this work, we summarized and described the major developments of modelling approaches in radical polymerization (RP) of MVMs and critically highlighted their applications on FRP and RDRP systems. This Minireview, therefore, serves as a concise introduction to push the boundaries of using modelling techniques in MVMs polymerization study.

## 2 Modelling Techniques

A universal kinetic model can provide guidance for the synthesis of tailored polymer architecture with desired properties. So far, a number of modelling methods have been developed to study radical (co)polymerizations of MVMs, which can be classified into three predominant types—statistical models,[^1^, ^11^, ^12^, ^13^] deterministic method (kinetic‐based models)[^14^, ^15^, ^16^, ^17^, ^18^, ^19^] and stochastic approaches (Monte Carlo).[^20^, ^21^, ^22^]

**Figure 2:**
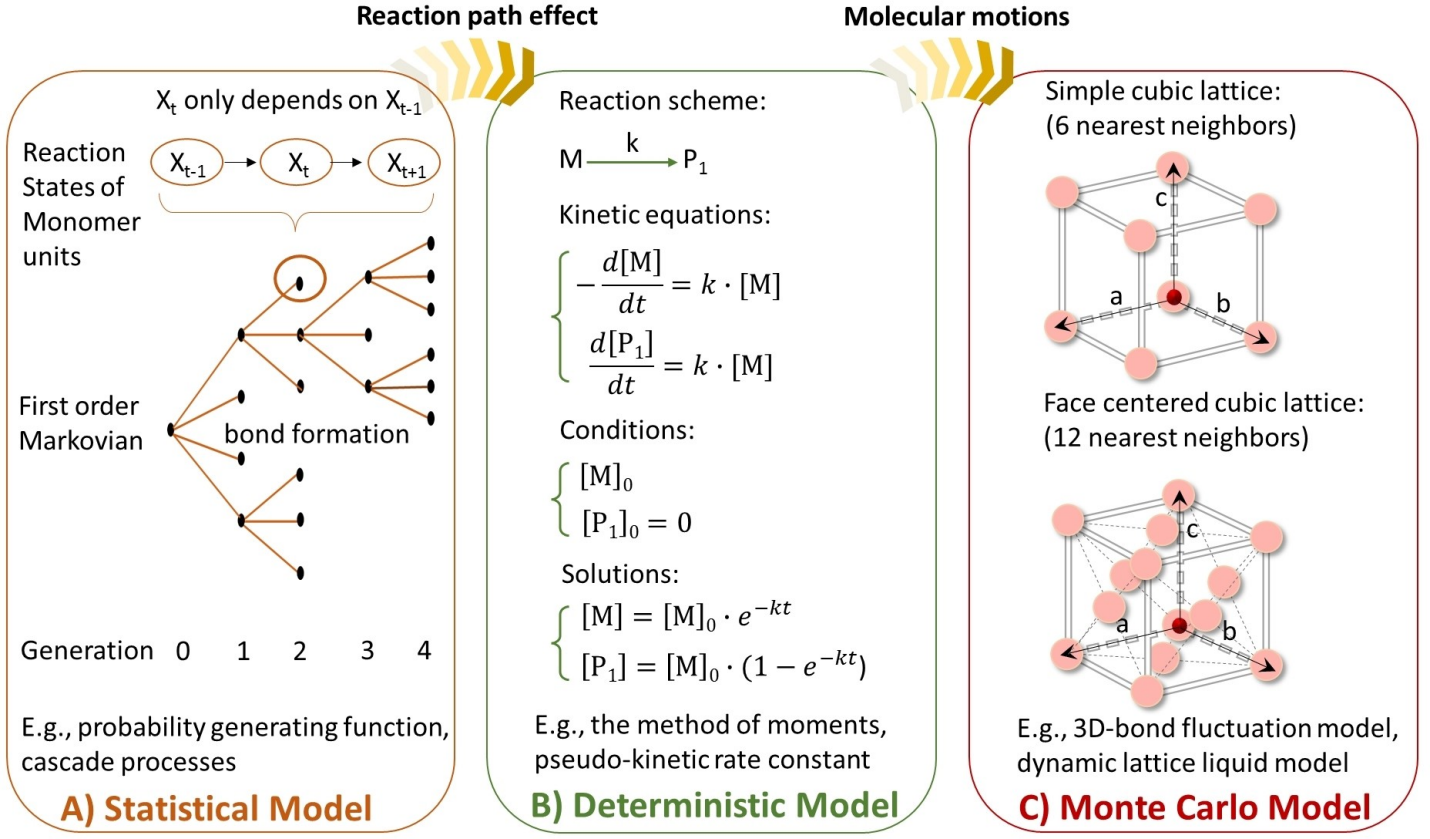
Representation of three theoretical modelling methods (statistical method, kinetic modelling, and Monte Carlo simulation) for the description of network formation process in RP of MVMs.

The statistical model (Table 1) is the oldest modelling method for studying RP of MVMs. Following the bond formation rules, the branched or crosslinked structure is generated from units in different reaction states, and various average properties for the macrostructure can be obtained (Figure 2A).^[13]^ However, the statistical models do not consider the effect of kinetic path or reaction history. The deterministic approach (Table 1) is a kinetic‐based modelling technique, in which kinetic equations or numerical approaches would be used to gain an insight into the mechanism of various polymerization processes.^[2]^ Nevertheless, the deterministic approach usually involves the simultaneous solution of algebraic/differential equations derived from the mass balances. For a large system, this solution is rather difficult and computationally expensive. To simplify the simulation system, the method of moments,^[23]^ numerical fractionation (NF) technique,^[15]^ pseudo‐kinetic rate constant method,^[16]^ and commercial software^[17]^ etc were developed by, for example, properly applying simplifying assumptions, or grouping chain lengths into finite intervals (Figure 2B). Unlike these kinetic‐based approaches, the stochastic approach (Table 1) is achieved by solving the chemical master equations.^[24]^ Reactants are allowed to distribute in different reaction states and gradually form the macrostructure. Using this method, one does not have to derive and solve a set of differential equations, only the knowledge of reaction probabilities is required. Therefore, the stochastic‐based approach is especially applicable to complex polymerization systems.

**Table 1:** Various modelling methods for RP of MVMs and their application scopes.

Scheme		Characteristics	Authors	Ref.	Applications
(1)	Statistical models	» Tree‐like structure generated from units in different reaction states » First order Markovian Bond formation rule	Flory and Stockmayer	[1]	• Gelation process, gel point calculation • Effect of cyclization (multiple crosslinking) • Average structural properties prediction (MW, cyclization, termination, and gel effect etc)
			Dusek and co‐workers	[11]	
			Macosko and co‐workers	[12]	
			Peppas and co‐workers	[13]	
(2)	Deterministic (kinetic based) method	» Solution of kinetic differential equations			• Cyclization constants • Pendant reactivity • Network formation
	• The method of moments		Bamford and Tompa	[14]	• Crosslinking kinetics • Gel point prediction • Crosslinker feeding strategy • Extent of branching and cyclization
	• Numerical Fractionation		Teymour and Campbell	[15]	• Cyclization kinetics • Gelation dynamics • Chain length distribution (CLD)
	• Pseudo‐kinetic rate constant		Hamielec and Tobita	[16]	• Crosslink density • Gel point prediction
	• Predici		Wulkow	[17]	• Gel point prediction
	• Multifunctional Polymer Molecule (MFM)		Vivaldo‐Lima and co‐workers	[18]	• Effect of crosslinker on gelation process and network morphology • Branching degree prediction
	• Generating Functions (GF)		Dias and co‐workers	[19]	• Effect of pendant reactivity on polymer structure • Cyclization kinetics
(3)	Stochastic models (Monte Carlo)	» Probability‐based » Solution of chemical master equations			• Polymer structure homogeneity • Primary chain distribution • Network formation • Evolution of MW, CLD, polymer composition
	• Gillespie algorithm		Gillespie	[20]	• Gel point prediction • Effect of intramolecular cyclization • FRP and RDRP crosslinking process pre‐ and post‐ gelation • Molar mass distribution between crosslinks • Complete CLD prediction
	• Lattice model		Manneville and De Seze	[21]	• Polymer structure inhomogeneity • Intramolecular cyclization (rate, extent etc) • Gel formation and properties (swelling behaviour)
	• Monte Carlo Sampling		Tobita	[22]	• Formation processes of branched or crosslinked polymers

Monte Carlo, the most widely used stochastic technique, was born in 1944 with the aim to simulate the diffusion of neutrons in fissionable materials.^[25]^ Considering the complexity of the chemical architecture and the dimensionality of the geometrical structure of macromolecules, it becomes prohibitive or even impossible to give a successful prediction using deterministic or nonstochastic approaches.^[26]^ However, via Monte Carlo, no matter how complex the systems are, they can be described as a series of states occurring in a stochastic manner under specific reaction probabilities.[^27^, ^28^, ^29^] Naturally, Monte Carlo becomes an important and invaluable tool perfectly adaptable to the stochastic and discrete nature characterizing problems in the field of polymer science. Notably, Monte Carlo method relies on the use of random numbers, thus it requires high quality random numbers to obtain a reliable prediction, and the selection of an appropriate random number generator is essential. Furthermore, given the differences of the random numbers generated in each run, properly tuning the system size is also required for accurate results.

Depending on the nature of the polymerization system and the targeting outputs, different variations of Monte Carlo algorithms may be adopted (Table 1).^[29]^ Specifically, regarding RP of MVMs, the two most commonly used algorithms are Gillespie's algorithm and lattice algorithm. Gillespie first introduced an exact stochastic simulation method for spatially homogeneous chemical systems (also called the stochastic simulation algorithm (SSA)) based on a function of reaction probability density.[^2^, ^20^] It has become the most widely used technique for the simulations of RPs by numerically calculating the time evolution of a molecular mixture which reacted through a series of stochastic reaction channels. It has been repeatedly verified as a powerful tool to study not only the average properties of macromolecular chains, but also the full MWD. The behaviour near the gel point in polymer science is similar to the critical behaviours in percolation problems.^[30]^ Thereby, an important class of Monte Carlo algorithms regarding the RPs of MVMs is the lattice algorithm. In 1981, lattice Monte Carlo has been used in multifunctional polymerization,^[31]^ as a tool to allow for loop formation and excluded volume effects (both of which are negligible in off‐lattice algorithms). Since then, the lattice models have become a powerful tool in studying the MVMs polymerizations where the effect of cyclization, diffusion or slow relaxation can be involved (Figure 2C).

## 3 Application

**Figure 3:**
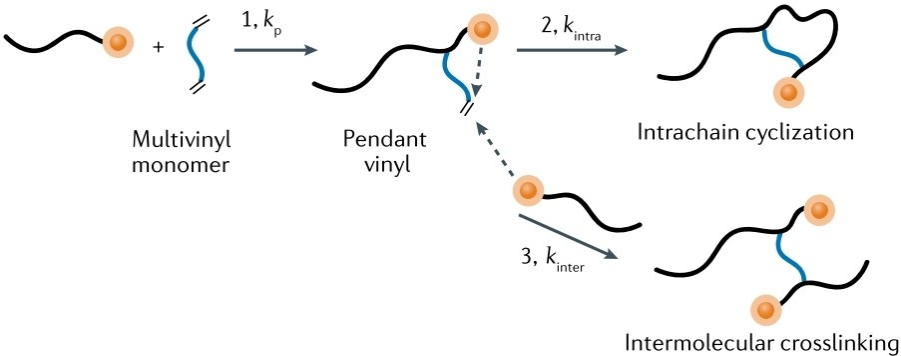
Features of RP of MVMs. The RP process of MVM possesses unique mechanistic processes and special reaction kinetics, originating from the generation of polymerizable pendant vinyl moieties on the growing chain (reaction 1), and these pendant vinyls can continue to react with propagating radicals through either intrachain cyclization (reaction 2) or interchain crosslinking (reaction 3). Reprinted with permission from (Ref. [32]). Copyright (2020) Springer Nature Limited.

The RP of MVMs possesses unique mechanistic processes originating from the generation of pendant double bonds (PDBs) on the growing chain. The growing chains can react with monomers, and also with the PDBs through either intrachain cyclization or interchain crosslinking (Figure 3). Modelling techniques are used primarily to understand the special reaction kinetics of these processes, while offering the advantages of avoiding tedious experiments.

### 3.1 Conventional Free Radical Polymerization

In 1940s, Flory^[1]^ and Stockmayer^[33]^ proposed the first gelation theory (F‐S theory) based on the mean‐field statistical model. It predicted that the polymerization of MVMs inevitably leads to gelation even at low monomer conversion (<10 %) by employing two assumptions: (1) Intramolecular cyclization can be neglected (in concentrated conditions); (2) Equal reactivity of all functional groups. These assumptions greatly simplify the mathematical analysis of the model but also result in discrepancies between the gel points from F‐S theory and experiments. Since then, more advanced models were applied to the FRP of MVMs systems to investigate their crosslinking behaviour. Pandey and co‐workers,[^34^, ^35^] for example, revealed the polymer structure inhomogeneity that deviates from the random growth (i.e., F‐S model) utilizing the Monte Carlo kinetic gelation lattice model.^[21]^ Tobita and Hamielec[^36^, ^37^] further investigated the inhomogeneity phenomenon by developing a kinetic model based on the pseudo‐kinetic rate constant method, and demonstrated that the inherent inhomogeneity of crosslink density of primary chains theoretically vary with their different born time. Furthermore, they correlated this inhomogeneity with the unequal reactivities of double bonds and/or substantial cyclization.^[38]^

In terms of intramolecular cyclization, the symmetric isotopic labelling disassembly spectrometry (SILDaS), developed by Johnson and colleagues,[^39^, ^40^, ^41^, ^42^] is an outstanding model‐free strategy for directly quantifying intrachain connections in end‐linked and side‐chain‐crosslinked polymers. However, to the best of our knowledge, the application of this technique to MVM‐derived polymers has not yet been reported. Towards the intrachain linkages in RPs of MVMs, the lattice model exhibits obvious potential to show its extent.^[43]^ Anseth and Bowman^[44]^ improved the traditional simple cubic lattice model to a face centered cubic lattice, and found that the high localized concentration of PDBs leads to increased primary cyclization, and that the distance between PDBs controls the cyclization rate. In addition to the stochastic method, Okay et al.[^45^, ^46^, ^47^, ^48^] established a kinetic model (deterministic method) for the FRcP of 1,4‐divinylbenzene (DVB) and styrene to investigate the cyclization in dilute conditions (monomer concentration <5 w/v%). In their study, the cyclization degree reached above 60 % as the dilution increased. The higher primary chain length or the cross‐linker proportion could also promote the cyclization. Besides, due to the extensive cyclization, the average reactivity of PDB for intermolecular crosslinking was 2–3 orders of magnitude lower than that of the free vinyls. Following Okay's methodology, the effects of solvent on the cyclization kinetics in FRcP of styrene and DVB were modeled by Aguiar et al.[^49^, ^50^] using the NF technique. They reported that the cyclization is more favored in reaction systems containing a poor solvent than a good solvent, suggesting the more the chain is stretched out, the lower the intrinsic rate of cyclization.

**Figure 4:**
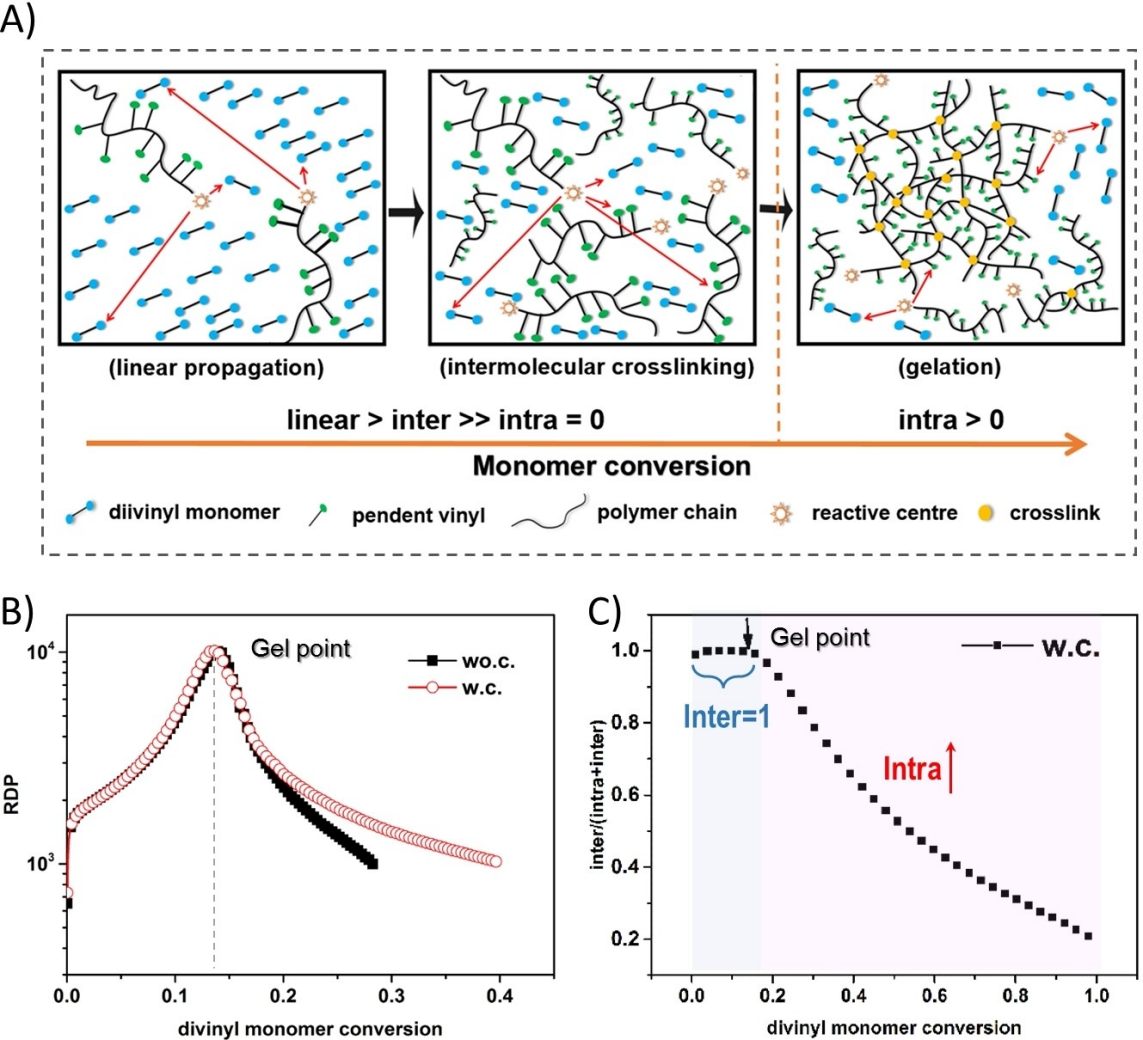
A) Illustration of the three polymerization stages in FRP of MVMs. B) The growth of reduced degree of polymerization (RDP) with divinyl monomer conversion. The peak indicates the gel points. C) Ratio of intermolecular crosslinks per chain to all crosslinks per chain versus divinyl monomer conversion. Readapted with permission from (Ref. [51]). Copyright (2017) Science China Press and Springer‐Verlag GmbH Germany.

The more and more results reported about the effect of cyclization on FRP crosslinking processes have led to a debate regarding the applicability of F‐S theory in FRP of MVMs. Nevertheless, those reported theoretical models are more or less deviated from the exact F‐S theory, either taking into account the dimension and spatial coordinates of the reactants or making the reactivity of functional groups unequal. To clarify whether F‐S theory is suitable for predicting FRP, and to find out to what extent cyclization participates in FRP of MVMs, thus elucidating the mechanism of FRP of MVMs, Wang et al.^[51]^ recently simulated FRP/FRcP of divinyl monomers (Figure 4A). The simulation was based on two kinetic models—with cyclization model (**w.c**.) and without cyclization model (**wo.c**., corresponding to the F‐S theory) via Monte Carlo simulation (following Gillespie's algorithm). **w.c**. and **wo.c**. models are both statistical models, where a coarse‐grain approach was used, and the coordinates for all the molecules in the space were not taken into account. The reactivity of functional groups was assumed to be constant. The simulated results were compared with the F‐S theoretical value and the experimental results. It was found that both the **w.c**. and **wo.c**. models gave a correct prediction of the gel point (Figure 4B). This indicates that in a concentrated FRP system, no intramolecular cyclization happens before gelation (consistent with the F‐S theory), which only occurs beyond the gel point (Figure 4C).

### 3.2 Atom Transfer Radical Polymerization

**Figure 5:**
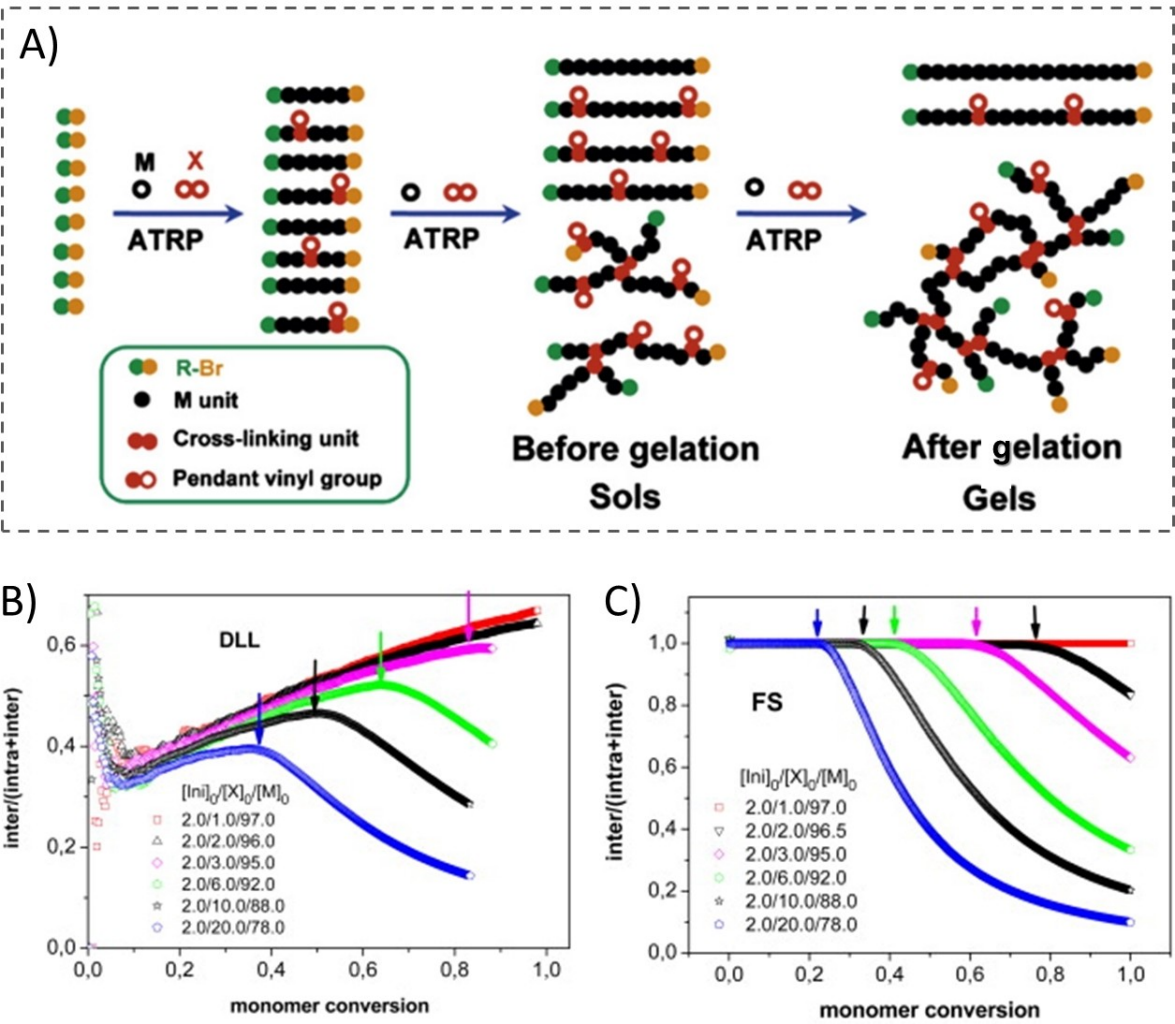
A) Illustration of the process of copolymerization of a monomer (M) and a cross‐linker (X) using ATRP technique (fast initiation) leading to gelation. Effect of an intramolecular cyclization in B) DLL and C) F‐S simulation. Ratio of intermolecular crosslinks per chain to all crosslinks per chain versus monomer conversion. Arrows indicate gel points. Readapted with permission from (Ref. [53]). Copyright (2010) Elsevier Ltd.

The branching and crosslinking in the ATRP process for acrylate monomers and diacrylate crosslinkers was studied experimentally and compared with Predici computations.^[52]^ The delayed experimental gelation (in comparison with the F‐S theory) was attributed to the unavoidable cyclization. Monte Carlo simulations provided more information on the ATRcP of vinyl and divinyl monomers[^9^, ^53^, ^54^, ^55^] using the off‐lattice and dynamic lattice liquid model (DLL, based on the cooperative movement concept. Excluded volume condition and integrity of polymer chains are observed. Polymerization reactions are simulated by forming new bonds between beads representing active radicals and monomers or crosslinkers with a predefined probability.) and Flory–Stockmayer (F‐S) models (Figure 5A). Nevertheless, different cyclization processes can be observed from the DLL model and the F‐S model. As shown in Figure 5B, the intramolecular cyclization continued during the entire ATRcP process in the DLL model (which predicted the gel points, MWD etc closer to the experimental values), while from the F‐S model (Figure 5C), the consumption of PDBs by intramolecular cyclization practically all occurred after the gel point was achieved.

**Figure 6:**
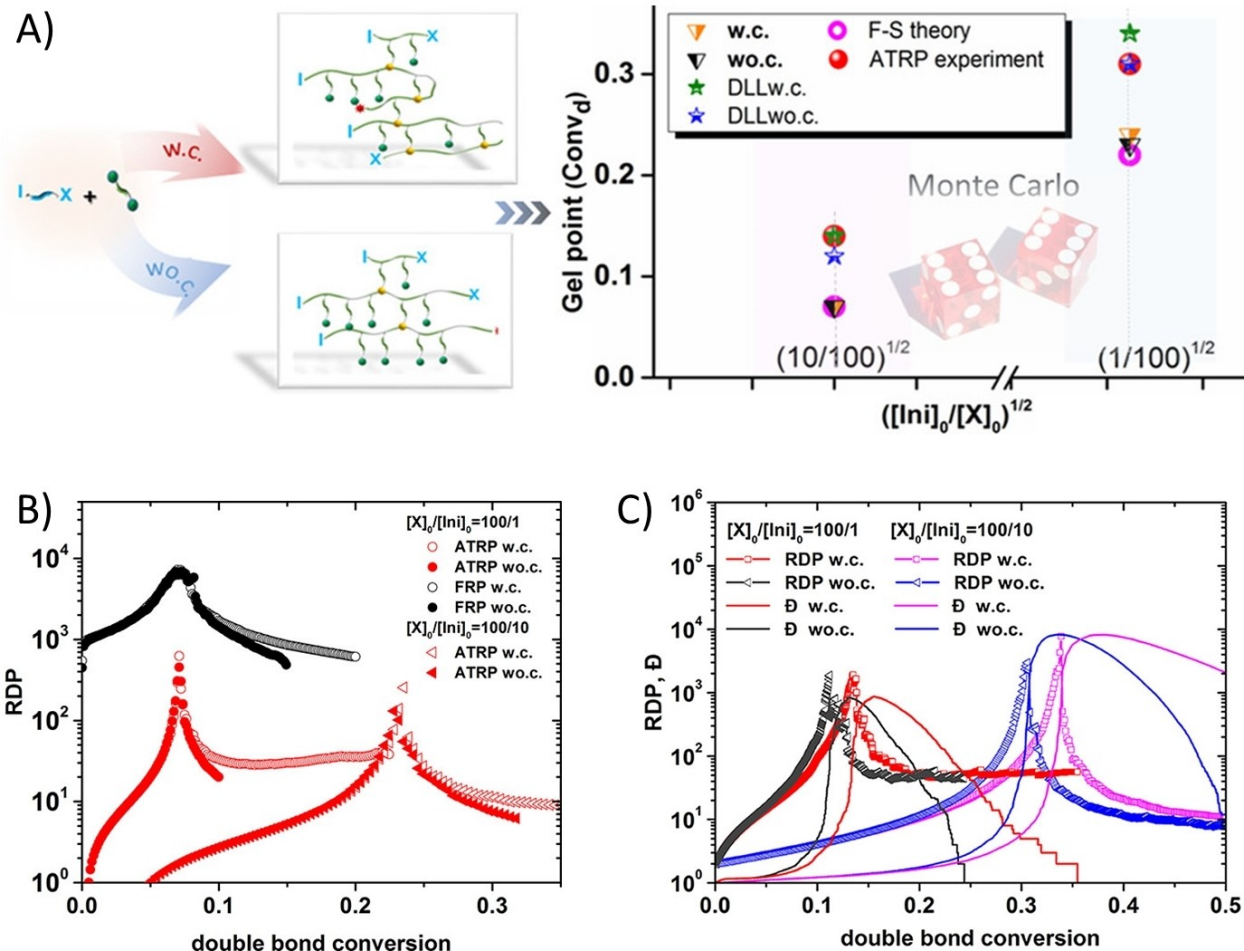
A) Monte Carlo simulations using two statistical models: with cyclization (**w.c**.) and without cyclization (**wo.c**., corresponding to F‐S theory), and DLL models in ATRP homopolymerization of divinyl monomers ([X]_0_). Comparison of the simulated gel points from **w.c**. and **wo.c**. models and those obtained from ATRP experiments, F‐S theory, and DLL models. B) Evolution of the reduced degree of polymerization (*RDP*) with double bond conversion, obtained from **w.c**. and **wo.c**. models of ATRP and FRP simulations. C) The changes in *RDP* and *Đ* with the increasing double bond conversion from DLL models. Readapted with permission from (Ref. [56]). Copyright (2018) American Chemical Society.

Recently, in relation to the homopolymerization of MVMs, the inapplicability and limitation of the F‐S model in describing the ATRP behaviour has also been reported by Wang et al.^[56]^ (Figure 6). Monte Carlo simulations using two statistical models **w.c**. and **wo.c**. (corresponding to F‐S theory), and DLL models were conducted to study ATRP of divinyl monomers. Their results demonstrated that the gel points obtained from both **w.c**. and **wo.c**. models were lower than the values from DLL models and experiments, indicating that the F‐S theory cannot be used to accurately predict the polymerization of divinyl monomers via ATRP. Furthermore, they demonstrated that the limitation of F‐S theory in predicting ATRP of divinyl monomers is not only due to the neglected intramolecular cyclization, but also due to spatial restrictions which cause the reactivity and accessibility of vinyl groups becoming non‐equivalent in ATRP of divinyl monomers.

### 3.3 Nitroxide‐Mediated Polymerization and Reversible Addition‐Fragmentation Chain Transfer Polymerization

**Figure 7:**
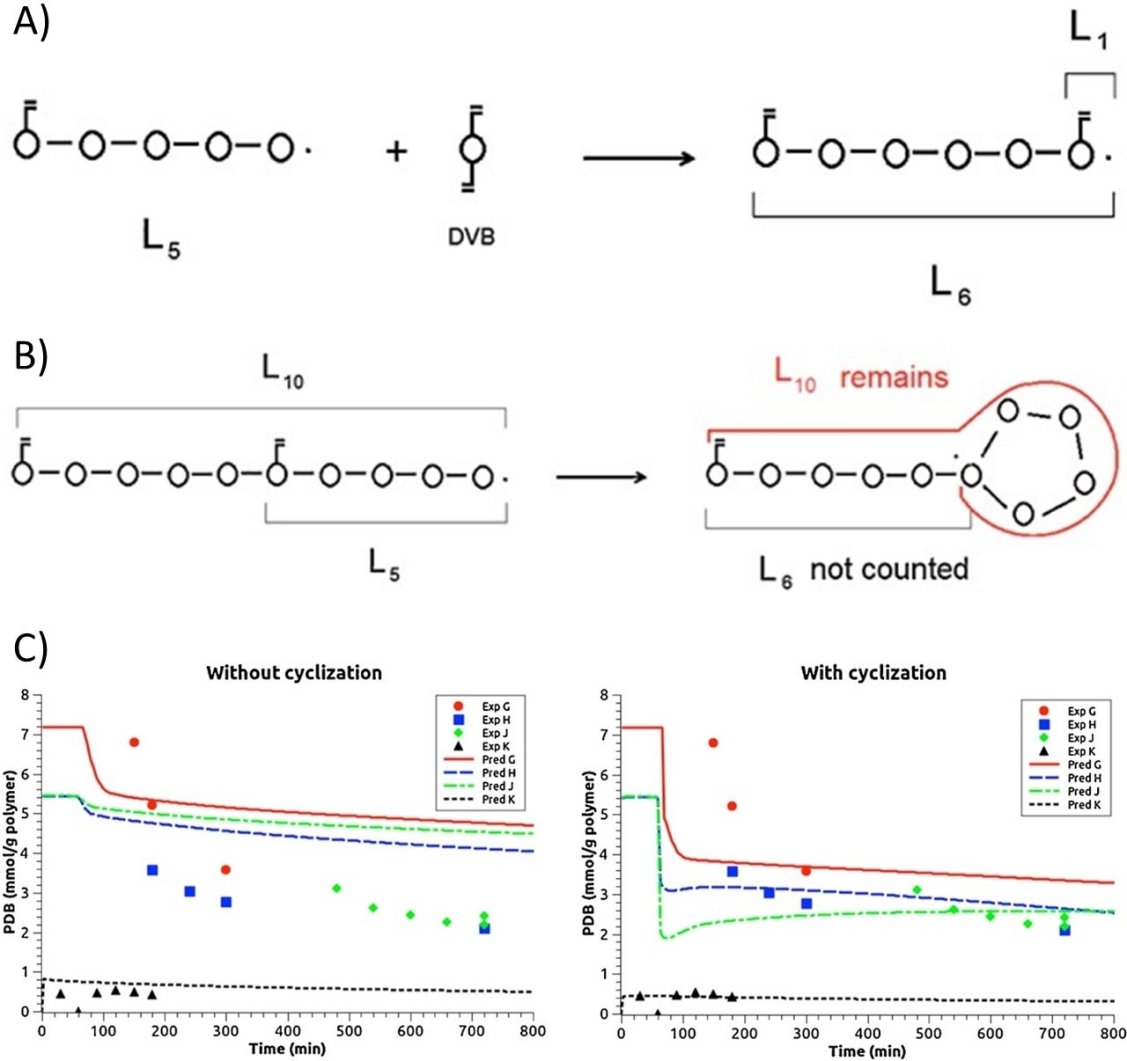
A) Schematic representation of the growing of a sequence with the simultaneous formation of a new L_1_. B) Pictorial scheme of consumption and creation of sequences due to cyclization. c) A comparison of the competition between crosslinking and cyclization reactions for the consumption of PDBs from without cyclization and with cyclization models. The monomer concentration in Exp H is 2.5 times that of Exp J. Readapted with permission from (Ref. [64]). Copyright (2013) WILEY‐VCH Verlag GmbH & Co. KGaA, Weinheim.

A series of modelling work on the kinetics of NMP crosslinking was conducted by Vivaldo‐Lima and Penlidis et al.[^18^, ^57^, ^58^] They first established a mathematical kinetic model for the NMP with crosslinking of styrene/DVB by using the method of moments (F‐S theory was used for the calculation of post‐gelation period), following a monoradical assumption.^[58]^ However, the monoradical assumption is actually unrealistic for the copolymerization systems of vinyl/divinyl monomers. Therefore, a more complete and realistic reaction model combining the MFM and the method of moments was further developed, in which several active and/or dormant radicals could simultaneously attach to a single macromolecule.^[57]^ Via the improved model, they proved the controllability of NMP of styrene/DVB in the presence of TIPNO‐based alkoxyamine (N‐tert‐butyl‐N‐(2‐methyl‐1‐phenylpropyl)‐O‐(1‐phenylethyl) hydroxylamine).^[18]^ The calculation efficacy of the MFM model in studying the kinetics of RAFT copolymerization of vinyl/divinyl monomers was also verified by Vivaldo‐Lima et al.[^59^, ^60^, ^61^] with either low or high crosslinker concentrations. Kinetic models describing NMP of styrene with DVB were also considered by Dias et al.[^62^, ^63^] Their simulations were based on a general kinetic approach using the population balances in terms of GF, where two isomers of commercial DVB (*m*DVB, *p*DVB) with different PDB (*m*PDB, *p*PDB) reactivities were taken into account. It was shown that the reactivity of PDBs controlled the structure (non‐linear) formation. Compared to the monomer vinyl groups, reduced reactivities of PDBs were obtained due to neglecting the effect of intramolecular cyclization. To understand the kinetics of intramolecular cyclization, Dias et al.^[64]^ then involved a balance of sequences of units (which connect a radical and a PDB present in the same polymer chain) in the kinetic model for NMP of styrene/DVB. In this more rigorous model, the cyclization rate constant was included as a function of the sequence length—$k_{Pij,r}^{C}$ (*i* and *j* are the type of radical and PDB, *r* is the sequence length) (Figure 7). In contrast to the model without cyclization involved, the improved model gave prediction results closer to the experimental data (Figure 7C). Moreover, the more favoured intramolecular cyclization over intermolecular crosslinking was observed in more diluted medium (Exp J *vs* Exp H in Figure 7C) due to the farther distance of the active centre from PDBs on other chains. Leiza et al.^[65]^ also quantitatively studied the NMP of styrene and DVB, but using a Monte Carlo method with the Gillespie algorithm implemented. The simulation quantitatively represented a more homogeneous polymer structure in NMP reflected by the narrower distribution of the molar mass between crosslinks (*M*_c_) compared to the FRP system. However, this model cannot be utilized as a quantitative measurement on cyclization, given the simplification of geometrical and accessibility constraints.

**Figure 8:**
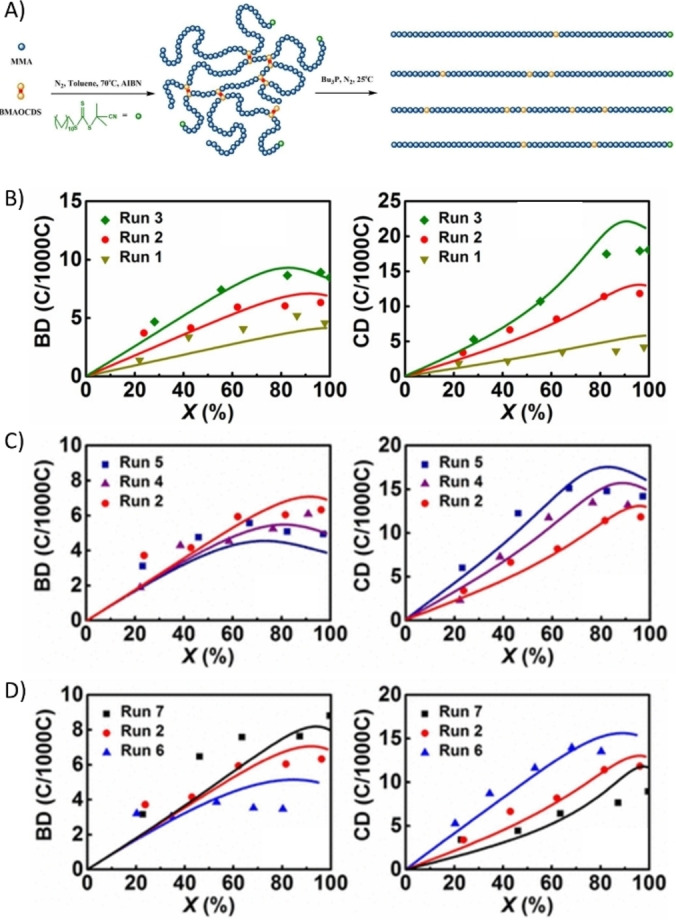
A) Synthesis of b‐PMMAs via RAFT copolymerization of MMA and BMAODS and their degradation to primary chains. Number‐average BD and number‐average cyclization density (CD, calculated based on BD) at various B) [MMA]_0_/[BMAODS]_0_ (run 1=50/0.5, run 2=50/1, and run 3=50/1.5); C) primary chain lengths [CTA]_0_/[MMA]_0_=1/50 (run 2), 1/75 (run 4), and 1/100 (run 5); D) initial monomer concentrations of 15 wt % (run 6), 30 wt % (run 2), and 45 wt % (run 7). Curves are model predicted values. Reprinted with permission from (Ref. [69]). Copyright (2016) American Chemical Society.

Regarding the crosslinking behaviour in RAFT system, recently, Matyjaszewski et al.^[66]^ conducted a systematic experimental comparison between networks made by RAFT and ATRP by utilizing cleavable crosslinkers. They found that at a low chain length, RAFT and ATRP produce comparable networks, while at a higher chain length, ATRP maintains better control. Modelling efforts have been made by Zhu and co‐workers to understand the RAFT crosslinking process. For example, Zhu et al.^[67]^ studied the gel points of RAFT copolymerization of vinyl/divinyl monomers based on the method of moments. The pseudo‐kinetic rate constant method was used to simplify the treatment of the copolymerization model. It was demonstrated that the classic Flory's gelation criterion (the onset of gelation is when the weight‐average number of crosslinked units per primary chain approaches unity)^[1]^ was valid for the RAFT gelation process if no intramolecular cyclization occurred. Zhu and co‐workers^[68]^ then extended the similar treatment to the modelling of semi‐batch RAFT copolymerization of acrylamide (AM) and N,N′‐methylenebis(acrylamide) (BisAM). Guided by the model, the constant feeding rate of BisAM was evaluated to produce hyperbranched structure free of gelation until complete monomer conversion. With the help of the above kinetic modelling method, the extent of branching and cyclization in RAFT crosslinking of MMA and a small amount (1–3 mol %) of cleavable dimethacrylate (bis(2‐methacryloyl)oxyethyl disulfide (BMAODS)) was also investigated. On the one hand, the kinetic modelling results verified the experimental quantification of the branching density (BD), which was estimated by comparing branched polymeric MW with that of its primary chains obtained from the degradation of the disulfide on the branched polymers (Figure 8A). On the other hand, it reported that in the above system, over 50 % PDBs were consumed via intramolecular cyclization instead of branching, and the intramolecular cyclization will be promoted with increased BMAODS ratio (Figure 8B), primary chain length (Figure 8C) and decreased monomer concentration (Figure 8D).^[69]^

## 4 Summary and Outlook

Important information, which is difficult or even impossible to access experimentally in RP of MVMs (such as the gelation process and polymer microstructure etc), have now been successfully obtained with the help of theoretical modelling techniques. Great progress has been made in terms of the polymerization kinetics and structural control, but the study of RP of MVMs to generate novel structured polymers and their formation mechanism is still in its infancy. Particular attention should be paid in future studies to the following aspects.

First, it is worth noting that despite numerous studies on FRP/RDRP of MVMs being published every year, controversies on their true mechanisms still exist (particularly regarding the homopolymerization system), which indicates that further studies based on both more advanced modelling and more detailed experiments are required to systematically study their essential difference in polymerization behaviours and to unify the mechanism understanding of FRP/RDRP of MVMs.

Second, the theoretical framework for the kinetically controlled polymerization of MVMs and synthesis of novel structured polymers (e.g., cyclized/ knotted single‐chain polymers) should be further studied. Various modelling techniques, such as Monte Carlo based on the lattice algorithm could be utilized to establish new advanced kinetic models towards the controlled MVMs polymerization process to reveal the novel structure evolution. Such models could consider the spatial restriction and chain relaxation of non‐Markovian chains. Molecular dynamics could also be utilized to calculate the anisotropic propagation probabilities.

Third, the relationship between reaction conditions and polymeric architecture (linear, branched, cyclized/knot and crosslinked network etc.) from RDRP of MVMs should be established. The effect of key parameters, such as the kinetic chain length, the feeding ratio of monomer and initiator, monomer concentration and reactivities etc. on the final structure of the produced polymers needs to be appropriately simulated and elucidated, which will serve as a theoretical guidance for the design and synthesis of complex polymers with tailored structure and functionality towards different future applications. In particular, the understanding and ability to manipulate intramolecular cyclization and intermolecular crosslinking within the homopolymerisation process of MVMs will be a revolutionary concept in polymer science.

Fourth, a universal gelation equation should be developed. On the basis of the new mechanism understanding of RDRP of MVMs, the classic F‐S theory is not suitable for the prediction of the RDRP of MVMs, thus the extension of this theory to a universal formula should be able to give appropriate predictions of the gel points in both the conventional FRP of MVMs and RDRP of MVMs, and applicable for both cases—with or without intramolecular cyclization. Moreover, possible sources of errors and improper uses should be pointed out to re‐establish precisely the validity and limits of the F‐S theory.

This review presents our understanding of the polymerization systems from the modelling point of view. It can be expected that with the trend towards increasing interest in studies on FRP/RDRP of MVMs, more and more advanced methodologies and breakthroughs will appear in the future. It is our hope that this review can assist interested researchers in utilizing modelling techniques for their investigations on polymerizations.

## Conflict of interest

The authors declare no conflict of interest.

## Biographical Information

*Jing Lyu received her B.Sc. (2015) in Materials Science and Engineering from North China Institute of Science and Technology, and M.Eng. (2018) in Materials Science from Tianjin University. In 2021, she completed her Ph.D. under the supervision of Prof. Wenxin Wang at University College Dublin. Since then, she has been carrying out Postdoc research in Prof. Wenxin Wang's group. Her research focuses on the mechanism study of novel structured multifunctional polymers based on experimental design and computer simulation*.

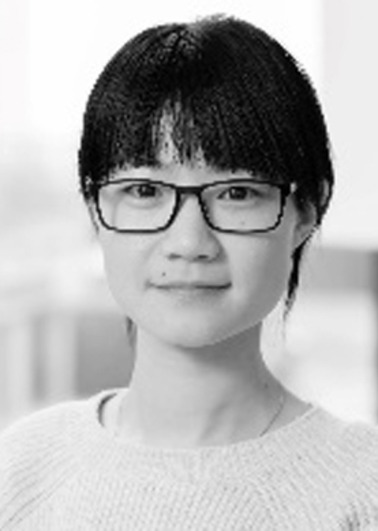

## Biographical Information

*Piotr Polanowski received his M.Sc. degree in physics in 1987 from the University of Lodz. He earned the Ph.D. in Chemistry from the Technical University of Lodz in 2002 and habilitation (D.Sc.) in Physics from the Adam Mickiewicz University of Poznan. He currently works in the Technical University of Lodz in the Department of Molecular Physics. His field of interest covers simulations of complex molecular and macromolecular systems with proper dynamic behaviour, parallel computing (hardware and software) in application to complex molecular systems, simulation software development*.

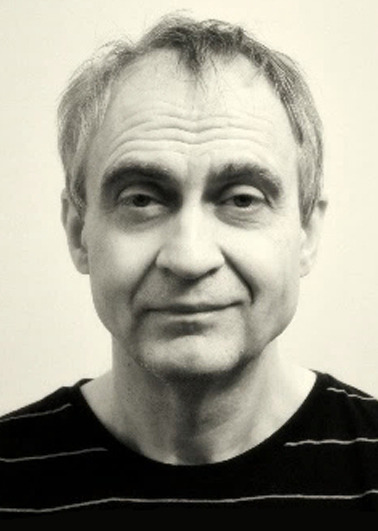

## Biographical Information

*Jeremiasz K. Jeszka received his master's degree in Physics in 1972 from Lodz University, his Ph.D. in 1978 from Lodz University of Technology and the title of Professor in Chemistry in 2008. His scientific interest concerns physical properties of polymers, polymer composites and interfaces. His early work was devoted to manufacturing electrical and magnetic properties of fully organic polymer composites, reticulate doped polymers. Later on he worked on preparation and properties of polymer composites with metal nanoparticles, carbon nanotubes and the effect of nanostructure of the solid surface on crystallization of the adjacent layer of polymers. He is also involved in Monte‐Carlo simulations of synthesis and properties of complex macromolecules and polymer brushes. Now retired*.

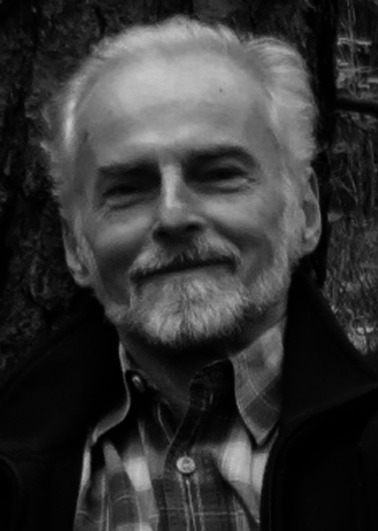

## Biographical Information

*Krzysztof Matyjaszewski is J. C. Warner University Professor of Natural Sciences at Carnegie Mellon University. His research is focused on synthesis of well‐defined macromolecules and hybrid materials via controlled polymerizations to prepare advanced materials for optoelectronic, biomedical, environmental and energy‐related applications. In 1994 he discovered Cu‐mediated atom transfer radical polymerization, commercialized in 2004 in US, Japan and Europe to prepare various advanced materials. He received 2021 Grand Prix de la Maison de la Chimie, 2017 Benjamin Franklin Medal in Chemistry, 2015 Dreyfus Prize, 2011 Wolf Prize in Chemistry and 2009 Presidential Green Chemistry Challenge Award*.

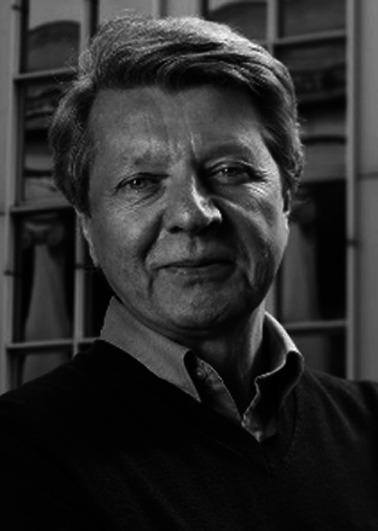

## Biographical Information

*Wenxin Wang is a Full Professor in Skin Research and Wound Healing at the Charles Institute of Dermatology, School of Medicine, University College Dublin (UCD), and “Yangtze” Scholarship Professor at the School of Public Health, Anhui University of Science and Technology. His research focuses on the development of multifunctional polymeric biomaterials, stem cell and gene therapy for skin wound healing. He won the “Young Scientist Prize in Regenerative Medicine” in 2010 at TERMIS‐EU conference, the “Science Foundation Ireland (SFI) Principal Investigator award” in 2011 and the DEBRA Award for Excellent EB Patient Service in 2014*.